# Recto-Vaginal Fistula

**Published:** 1878-05

**Authors:** Charles G. Stockton

**Affiliations:** Resident Physician


ART. II.—*Recto-Vaginal Fistula.*—Operation at Buffalo General Hospital, by Prof. J. F. MINER, M. D. Reported by CHARLES G. STOCKTON, M. D., Resident Physician.

On the 8th day of January, 1878, Mrs. L—— became a patient in the Buffalo General Hospital, to be treated for recto-vaginal fistula. She was American, 41 years of age, for some time past a widow, and had been occupied as house-keeper. Something less than six years since she became for the first time pregnant, and at the expiration of the full period was delivered, after the employment of forceps, of a male child of ordinary size. A perfect history of the delivery was not obtained, but the perineum was ruptured and measures were not taken to correct it. The general condition of the patient had always been good, but subsequently to her injury she suffered from involuntary discharges of gas and fæcal matter except when her bowels were kept confined.

On the date of her admission she appeared at Prof. Miner's clinic, and the examination exposed a fistulous opening, resolving the vagina and rectum into a single canal, extending from near the *cul de sac* of Douglass outwards through the entire septum. Before proceeding with the operation the Professor addressed the students, to the effect that he was very glad to have an opportunity of presenting one from a class of cases of great interest, and interesting indeed was the history of vesico-vaginal and recto-vaginal fistulæ, and of the operative procedures invented for their alleviation; conditions that must have arisen in all ages, and only recently completely remedied, and even now the perfect cure of some varieties was a matter of doubt, and such a case was that now present. The matter had been long discussed, but no reasonable treatment was suggested until the sixteenth century, and nothing like success was accomplished until 1864, when our own countryman, Prof. Sims, announced an operation which, by certain discoveries and improvements over the methods of others, was said to contain for the first time the essentials of success. The difficulties were to expose the fistulous tracts to view, to render them available to approach and operation, and to protect the parts from the irritating excrements that must find an exit. These difficulties were met and overcome in manners varying with the extent and character of the affections, and patients were relieved of the most uncomfortable conditions. Fæcal fistulæ were the first to receive surgical assistance, but contrary to what might have been expected the best results were not obtained upon this affection. Text-books contained ample directions, and journals reported flattering accounts of operations, which, in point of fact, were only comparatively successful.

You are to remember that these cases were, some of them improved, some impaired, and others probably cured. In the reports of surgical manipulations it too frequently happened that the *successes* only were reported, and not the failures. You are to understand that not alone the general practitioner would have unfavorable facts to record regarding these cases, but also those who were termed experienced surgeons; some of the very best having failed to make the lacerated septum grow together, and it is even more difficult to obtain union where the fistula arises from an ulcerating or specific condition. In a case very similar to that under consideration the unhappy patient had been seven times operated upon without material improvement, and at this stage the case came into the Professor's hands. As the previous efforts were made by skillful men, after the usual method and with slight advantage, he devised a new operation, and for the first time put it to trial. It consisted in cautiously dissecting from its attachments the lower end of the rectum, seizing it and drawing it down so that the upper part of the fistulous opening corresponded to the surface of the verge of the anus. The rectum was severed at this point, and the extremity stitched carefully to the integument of the adjacent parts ; thus securing a new septum of sound, uncicatrized tissue, dispensing with all efforts to obliterate the fistula. The result of this operation was very gratifying, both to patient and surgeon, and he believed the suggestion might yet prove valuable.

Of the case now waiting for cure, he explained that the separation was very complete and extensive, and he would attempt to correct it by the ordinary method. Anæsthesia was procured by the administration of pure vapor of ether, and the patient, with very few struggles, soon became insensible to pain. Her position was that commonly employed in lithotomy; an assistant on either side steadied the knees and held apart the labia, while a third lifted the anterior vaginal wall by means of a Sims' speculum. The septum was now well exposed and thus held by the hooks, while the edges of the fistulous tract were pared by a sharp bistoury, and the margins were approximated and retained by interrupted wire sutures placed closely together.

This constituted the first operation, the closing of the perineum being left for another occasion. A catheter was introduced and the bladder evacuated frequently. On the day following the woman was quite cheerful and painless; the next day her pulse had increased from 90 to 110 per minute, and her temperature from 98° to 101° Fah. The fourth day, Jan. 12th, she anticipated her expected period ten days. She also suffered from rigors, and what she had not previously experienced, general *malaise* attended the rise in pulse and temperature. The wounded parts were too sensitive and red; and that these were the *prodromi* of erysipelas was established by the bright blush that soon overspread the neighboring parts. The patient was removed to an isolated "Pavilion" ward, under the constant attention of a faithful nurse. Ventilation, temperature, diet and hygienic measures generally were carefully regarded; the sanguinous discharge was removed by frequent ablutions, and opium, which had been before used for the effect of constipation, was continued.

January 13th, pulse 160, temperature 105° Fah. Quinine and the Tr. of Cloride of Iron were prescribed by the house physician, and Iodine in tincture, was applied to the erysipelatous surface.

January 19th, pulse 110. temperature 100° Fah. She convalesced steadily, and an attempt was made to remove the sutures, January 18th, but the union was found too imperfect to allow it and the removal was delayed one week later.

February 13th it was first deemed expedient to make the second operation. Prof. Miner remarked that the inter-current disease had disturbed his plans; that the erysipelas appeared grave, involving the greater portion of the body, but she was now in fair physical condition, owing to what was usually denominated therapeutics, by *doctors*, but was in fact the natural tendency of the disease. He wished the class to go out to their work with a distinct knowledge that many diseases are self-limited, and markedly so erysipelas. The exposure might be owing to the work of man, but its cure is entirely natural, perhaps uninfluenced by medicines. This patient was restored by removal to a ward that had on all sides free, pure air, by attentive nursing, ventilation and good diet. Since quinine and iron had been administered, he could not prove that in this case they had no particular effect, but he maintained that the progress of erysipelas depends not upon medication.

The patient having been etherized, the ruptured perineum was pared and brought evenly and accurately together, and deep quilled sutures used for retention. The urine was voided through a catheter, and the bowels kept constipated for ten days, and then the expulsion was assisted by proper enemata. After this the woman considered herself as well as ever. At a final examination of the patient three weeks later, the operator remarked that this was one of his best results, and the repair seemed perfect, and the physiognomy of the parts perfectly natural.

